# Evaluation of a laboratory-based high-throughput SARS-CoV-2 antigen assay for non-COVID-19 patient screening at hospital admission

**DOI:** 10.1007/s00430-021-00706-5

**Published:** 2021-04-15

**Authors:** Friederike Häuser, Martin F. Sprinzl, Kim J. Dreis, Angelique Renzaho, Simon Youhanen, Wolfgang M. Kremer, Jürgen Podlech, Peter R. Galle, Karl J. Lackner, Heidi Rossmann, Niels A. Lemmermann

**Affiliations:** 1 Institute of Clinical Chemistry and Laboratory Medicine, University Medical Center Mainz, Langenbeckstr. 1, 55131 Mainz, Germany; 2 Department of Internal Medicine I, University Medical Center Mainz, Langenbeckstr. 1, 55131 Mainz, Germany; 3 Institute for Virology and Research Center for Immunotherapy (FZI), University Medical Center Mainz, Langenbeckstr. 1, 55131 Mainz, Germany

**Keywords:** SARS-CoV-2 antigen test, COVID-19, LIAISON, Evaluation, Screening

## Abstract

Several rapid antigen tests (RATs) for the detection of SARS-CoV-2 were evaluated recently. However, reliable performance data for laboratory-based, high-throughput antigen tests are lacking. Therefore and in response to a short-term shortage of PCR reagents, we evaluated DiaSorin's LIAISON SARS-CoV-2 antigen test in comparison to RT-qPCR, and concerning the application of screening non-COVID-19 patients on hospital admission. Applying the manufacturer-recommended cut-off of 200 arbitrary units (AU/mL) the specificity of the LIAISON Test was 100%, the overall analytical sensitivity 40.2%. Lowering the cut-off to 100 AU/mL increased the sensitivity to 49.7% and decreased the specificity to 98.3%. Confining the analysis to samples with an RT-qPCR result < 25 Ct resulted in a sensitivity of 91.2%. The quality of the LIAISON test is very similar to that of good RATs described in the literature with the advantage of high throughput and the disadvantage of relatively long analysis time. It passes the WHO quality criteria for rapid antigen tests and is characterized by particularly high specificity. The LIAISON test can therefore be used for the same applications as recommended for RATs by the WHO. Due to limited sensitivity, the LIAISON test should only be used for screening, if PCR-based assays are not available.

## Introduction

The gold standard to detect SARS-CoV-2 genomes during COVID-19 diagnostics is quantitative reverse transcription PCR (RT-qPCR) [1, 2]. Despite limited sensitivity and specificity, the easy-to-perform antigen tests, which provide rapid and possibly also less expensive results, are currently of great interest, especially when PCR reagents and plastic ware, as well as trained personnel necessary to reliably perform the PCR tests, are scarce or simply not available.

For 2 weeks in early December 2020, at the second peak of the SARS-CoV-2 pandemic in Germany, the number of hospitalized COVID-19 patients at the University Medical Center Mainz increased significantly. Due to a short-term shortage of PCR reagents, all patients (1636), who were admitted to the hospital without a known COVID-19 diagnosis and who did not show any COVID-19-suggestive symptoms, were tested with the LIAISON SARS-CoV-2 antigen test. The rationale for selecting this test was based on the following considerations: (1) The required instrumentation (LIAISON) was already in use for other applications in the laboratory and the assay reagents were available. (2) Due to automation the analysis of > 200 samples per day was possible with the available staff. (3) The assay data given by the manufacturer [clinical sensitivity 97.1% (95% CI: 85.5–99.5%)] on PCR-positive samples (within 10 days onset symptoms) and clinical specificity 100% (95% CI: 96.5–100%) [3] were favorable compared to other antigen tests [4–8], despite all caution concerning antigen tests for the planned application in general [9]. Positive antigen test results were confirmed subsequently by SARS-CoV-2 specific RT-qPCR. In-patients who developed symptoms suggestive of COVID-19 were also promptly investigated by diagnostic PCR. It was planned to keep the substitutional period using the antigen test as short as possible. The data on antigen testing in general and especially with regard to the planned application are scarce and the manufacturer’s own evaluation of the LIAISON SARS-CoV-2 antigen test was based on a very small data set [10]. Hence, we aimed to evaluate the assay used in the current emergency situation at the University Medical Center to generate a solid basis for decision-making in the future and to compare the LIAISON assay to rapid antigen tests (RATs), as recently two of latter tests have been competently evaluated by Osterman et al. [11].

## Materials and methods

### Patient collectives

#### Asymptomatic screening cohort

Between Nov 30 and Dec 14, 2020, all patients admitted to the University Medical Center Mainz without COVID-19 symptoms were screened for SARS-CoV-2 using the LIAISON SARS-CoV-2 antigen test (Diasorin S.p.A, Saluggia, Italy). All positive antigen tests were confirmed by RT-qPCR; furthermore, PCR testing was performed in patients with new-onset symptoms suggestive of COVID-19. The age of the evaluated patients ranged between 2 months and 95 years with a median of 59 years. 45.84% (*n* = 750) were female, 54.16% (*n* = 886) were male.

#### Evaluation collective

Between Dec 9, 2020, and Jan 29, 2021, 196 nasopharyngeal swabs of 139 COVID-19 patients were analyzed by the LIAISON SARS-CoV-2 antigen test. The age of the patients ranged from 21 to 97 years with a median of 71. 40.3% (*n* = 56) were female, 59% (*n* = 82) male. For one individual, the sex is not reported. All patients had been diagnosed with COVID-19 confirmed by SARS-CoV-2 PCR from the sample analyzed either in this study or from a previous sample. 133 of 139 patients were hospitalized due to COVID-19. Sample collection was performed at any time during the course of the disease. Due to convalescence, 27 samples were already tested negative by PCR. Multiple samples (2–6) were taken from 49 patients with intervals between samples of a few hours up to 19 d.

#### Sampling

Sampling was performed with the Σ-Virocult system (Medical Wire and Equipment Co Ltd, Corsham, England) by trained personnel. The time from sample collection to examination by PCR and antigen test did not exceed 24 h. Both tests were always performed on the same specimen and not on different swabs taken in parallel.

#### SARS-CoV-2 antigen assay

All sampled nasopharyngeal swabs in viral transport medium were processed with the SARS-CoV-2 Ag chemiluminescence sandwich-immunoassay (CLIA) on LIAISON (Diasorin) according to manufacturer instructions. 1 mL of transport medium was transferred to a tube with 1 mL inactivation buffer, vortexed, pre-incubated for 120 min (virus inactivation), and loaded on the instrument. Results (Light Units) of the CLIA are automatically recalculated and given as an equivalent to Tissue Culture Infectious Dose (TCID50/mL) by the instrument. As the manufacturer-specified cut-off for a positive test-interpretation is 200 TCID50/ml, and the limit of detection (LOD) is given as 22 TCID50/mL by the manufacturer, but most negative readings range between 40 and 110 TCID50/mL according to our experience, we decided to report the quantitative results of the antigen assay as arbitrary units (AU/mL).

#### SARS-CoV-2 RT-qPCR

##### Laboratory 1

Analyzed 110 nasopharyngeal swabs by RT-qPCR for SARS-CoV-2 RNA. Viral RNA was extracted and amplified from respiratory samples using the SARS-CoV-2 Test Strip on a NeuMoDx 288 (NeuMoDx Molecular, Inc., Ann Arbor, USA) according to manufacturer´s instructions detecting the SARS-Cov-2 genes N and Nsp2. Ct values of the reference samples [12] were as follows: Sample 1 (Ch07469) approximately 10^6^ copies/mL: N-gene: 22.0, sample 2 (Ch07470) approximately 10^7^ copies/mL: N-gene: 19.0

##### Laboratory 2

Analyzed 86 nasopharyngeal swabs by RT-qPCR. RNA was extracted with the QIAamp Viral RNA Mini Kit according to manufacturer instructions (QIAGEN GmbH, Hilden, Germany). A control DNA fragment (Equine Arteritis Virus (EAV); TIB Molbiol Syntheselabor GmbH, Berlin, Germany) was added to each sample prior to extraction. Oligonucleotides as well as the control fragments for the three targets were synthesized by TIB MolBiol and distributed as “LightMix”. One-Step QuantiTect Probe RT-PCR Kit was purchased from QIAGEN.

As suggested by Corman et al. [1], two RT-PCR assays were performed. The first-line assay detects the E-gene and the EAV extraction control. A positive assay was followed by the confirmatory reaction (RdRP-gene). Both assays were performed under the following conditions: the 25 μl reaction set up contained 10 μl RNA, 12.5 μl 2 × QuantiTect Probe RT-PCR MasterMix, 0.5 µl QuantiTect RT Mix, 0.5 µl LightMix (containing primer and probes for E-gene and EAV or RdRP-gene), and 1.5 µl water. Thermal cycling was carried out on a cobas z480 analyzer (Roche, Mannheim, Germany): 50 °C for 30 min (reverse transcription), 95 °C for 15 min and 45 amplification cycles of 94 °C for 15 s and 60 °C for 60 s.

Ct values of the reference samples [12]: sample 1 (Ch07469) approximately 10^6^ copies/mL: E-gene: 25.49, RdRP-gene: 25.12, sample 2 (Ch07470) approximately 10^7^ copies/mL: E-gene: 22.19, RdRP-gene: 21.63.

The use of anonymized, diagnostic surplus material for the evaluation and validation of diagnostic tests was approved by the local ethics committee (Ethik Kommission der Landesärztekammer Rheinland-Pfalz) and is part of the patient admission agreement (§ 14 Abs. 3) of the University Medical Center Mainz.

## Results

### Assay specificity

**Fig. 1:**
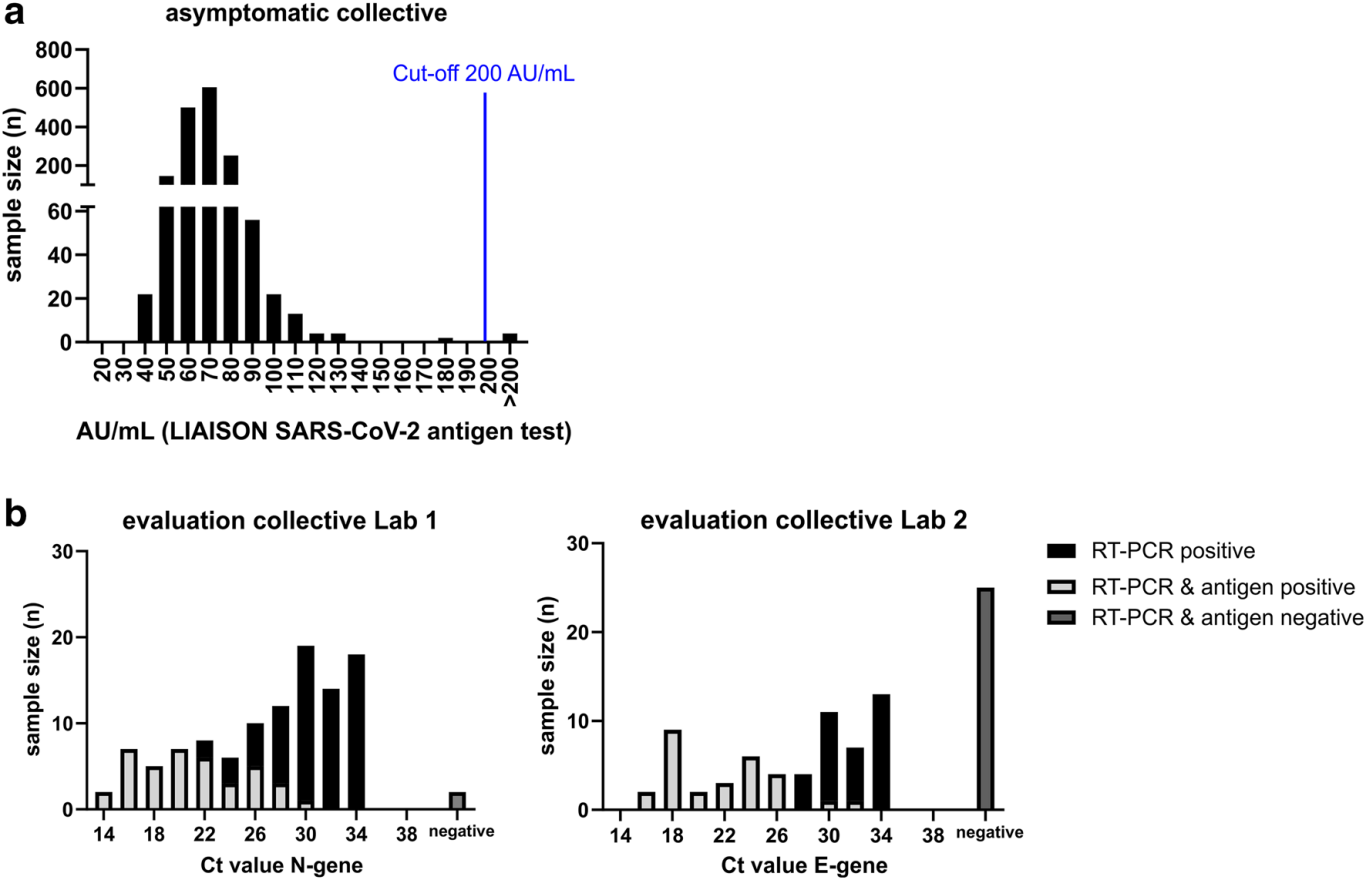
Sample number (n) according to (**a**) the quantitative results of the antigen test (AU/mL: arbitrary units; relative light units converted to TCID50/mL equivalents by the manufacturer) in the asymptomatic collective and (**b**) the quantitative results of the PCR (Ct) assay in the evaluation collective. 4 out of 1636 samples in the asymptomatic screening cohort (**a**) were tested positive (cut-off 200 AU/mL) by the antigen test and were all confirmed by PCR

1636 patients without COVID-19 symptoms were routinely screened for SARS-CoV-2 at hospital admission using the LIAISON antigen assay and were included in the analysis (Fig. 1a). During the 2 weeks of testing, the 7-day incidence in the city of Mainz, from which and its closer vicinity most patients originated, was 115.05–214.8 /100,000 inhabitants (49/2020 and 50/2020), according to official data of the Robert-Koch-Institute, Berlin [13]. 4 out of 1636 patients (0.24%) were tested positive for SARS-CoV-2 Ag (Fig. 1a) with an assay cut-off of 200 AU/mL (recommended cut-off by the manufacturer), 1632 patients were negative. The 4 positive results were confirmed by RT-qPCR. This corresponds to a specificity of 100% (Table 1). Since personal communication with the test manufacturer indicated that false-negative results might primarily occur in the range between 100 and 200 AU/mL, 3 samples at 120.23, 166.87, and 187.8 AU/mL, which were detected within the first days of analyses, were tested by RT-qPCR. The results were negative, and consequently, there was no obvious evidence of a large number of false negatives at that time.

**Table 1:** Assay sensitivity for the SARS-CoV-2 antigen test in the evaluation collective (predominantly samples of symptomatic (and PCR-positive) patients during hospitalization) as well as the specificity in the asymptomatic screening cohort

Cut-Off ≥ 200 AU/mL							
	*n*	Antigen positive	Antigen negative	Sensitivity [%]	95% CI	specificity [%]	95% CI
Covid19 patients	196	68	128	40.2	33.1–58.4	n.d	n.d
Ct < 25	57	52	5	91.2	81–96.2	n.d	n.d
25 ≤ Ct < 30	47	14	33	29.8	18.7–44	n.d	n.d
Ct ≥ 30	65	2	63	3.1	0.9–10.6	n.d	n.d
Negative	27	0	27	n.d	n.d	100.0	n.d
Screening patients	1636	4	1632	n.d	n.d		
Correctly identified		4	1632	n.d	n.d	100.0	99.7–100.0

Cut-Off ≥ 100 AU/mL							
	*n*	Antigen positive	Antigen negative	Sensitivity [%]	95% CI	Specificity [%]	95% CI
Covid19 patients	196	84	112	49.7	42.3–57.2	n.d	n.d
Ct < 25	57	54	3	94.7	85.6–98.2	n.d	n.d
25 ≤ Ct < 30	47	23	24	48.9	35.3–62.8	n.d	n.d
Ct ≥ 30	65	7	58	10.8	5.3–20.6	n.d	n.d
Negative	27	0	27	n.d	n.d	100.0	n.d
Screening patients	1636	32	1604	n.d	n.d		
Correctly identified		4	1604	n.d	n.d	98.3	97.5–98.8

Sensitivity and specificity are given for the cut-off of 200 AU/mL, as recommended by the manufacturer, as well as for the cut-off 100. Confidence intervals were computed using Wilson score interval [20]

*AU* arbitrary units; relative light units converted to TCID50/mL equivalents by the manufacturer. *n.d.* not determined

In a retrospective analysis of the data, the cut-off was tentatively set to 100 AU/mL resulting in 4 PCR-confirmed and 28 potentially antigen-positive subjects. Three patients had been negative by RT-qPCR (see above). There was no material left to verify the negative result of the remaining 25 samples by RT-qPCR. Assuming that most if not all of these patients were negative, lowering the cut-off decreases the assay specificity in the worst case to 98.3% (Table 1).

In 14 of the patients from the screening collective, an RT-qPCR requested by the attending physician became positive 0–44 days after antigen testing. 2 of these patients had previously been positive in the antigen test (418.6 and 12,273.0 AU/mL), and in 11 other patients (40.8–88.3 AU/mL), the interval between the samples for antigen and PCR test exceeded 6 days. However, in one patient, a negative result in the antigen test (81.3 AU/mL) was followed by a highly positive RT-qPCR result (Ct N-gene 16.4) on the next day. We cannot exclude incorrect sampling in this case or a rapid increase in viral load, because the samples could not be retested with the other method.

### Assay sensitivity

The sensitivity of the antigen test was assayed on 196 samples of 136 COVID-19 patients. In 169 of the 196 samples, SARS-CoV-2 RNA could be detected by RT-qPCR. RT-qPCR tests were either performed in laboratory 1 using the NeuMoDx method to amplify SARS-CoV-2 genes N and Nsp2 (110 samples; Fig. 1b. left panel) or by laboratory 2 using an in house test to amplify the SARS-CoV-2 genes E and RdRP (86 samples; Fig. 1b, right panel), while antigen tests were all performed on the same LIAISON instrument. Only 68 of 169 PCR-positive samples were also positive in the LIAISON antigen assay (40.2%; Table 1). Sensitivity was significantly higher when only PCR positives with a Ct value below 25 were considered (91.2%). As expected, increasing the considered Ct range is associated with a lower sensitivity (e.g. Ct < 30: 63.5%). Lowering the cut-off of the CLIA to 100 AU/mL increases the overall sensitivity to 49.7%, but decreases the specificity accordingly (Table 1).

**Fig. 2:**
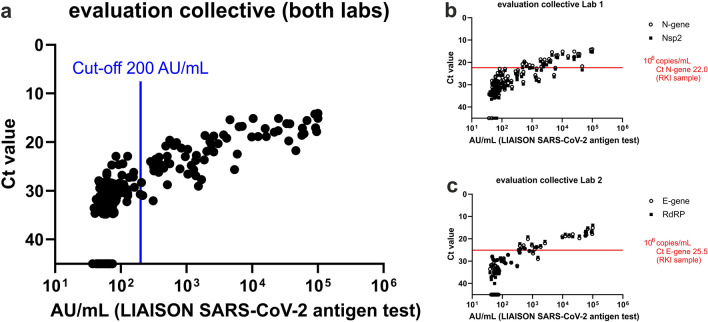
Detection of SARS-CoV-2 antigen in RT-qPCR tested nasopharyngeal swaps (negative PCR results are shown with a Ct value ≥ 45). Panel **a** compares the quantitative result of the antigen assay (AU/mL: arbitrary units) to the results of RT-qPCR carried out by two different laboratories (*n* = 196). Targets: N-gene for laboratory 1, E-gene for laboratory 2. **b** Laboratory 1 used the N-gene of SARS-CoV-2 as a screening assay and confirmed the result with the target Nsp2 (*n* = 110). The red line indicates the Ct value and the corresponding viral load of the reference sample of the Robert-Koch Institute for the screening target. **c** Laboratory 2 (*n* = 86): E-gene as screening target, RdRP as confirmatory target

Figure 2a demonstrates that there is also a quantitative correlation between the LIAISON antigen (AU/mL) and the RT-qPCR (Ct) results. This correlation was very similar no matter which of the two different RT-qPCR methods was used. The antigen test shows good sensitivity for Ct values up to 25 and significantly poorer sensitivity for Ct values ≥ 25 (Fig. 2b, c).

## Discussion

Until a large part of the population is vaccinated, the only option to avoid overwhelming the health care system in the COVID-19 pandemic (besides adhering to hygiene rules, wearing masks, and keeping a distance) is to test for SARS-CoV-2. In this respect, there is an urgent need for simple, rapid, and cost-effective testing methods in the diagnostic and clinical sector, but also in all areas of public life where people come together. This large amount of testing is difficult to achieve using only RT-qPCR, a highly sensitive and specific test system, which is a laboratory-based, laborious and expensive procedure requiring specialized equipment and well-trained personnel. In addition, the global demand for equipment, reagents, and plastic ware for PCR has already led to repeated shortages, which also affected the University Medical Center Mainz at the end of November 2020. The properties of rapid antigen tests (RAT) for point of care testing (POCT) have already been investigated in several studies, which have shown advantages (speed, on-site testing possible), but also major disadvantages in terms of test sensitivity and specificity as well as throughput [4, 11, 14, 15]. Therefore, the WHO does not recommend these tests for screening asymptomatic individuals in collectives with low COVID-19 incidence. Furthermore, in initial introduction of RATs into clinical use, WHO recommends careful evaluation of the RAT by comparison with PCR tests [9] to get a reasonable estimate of the test performance and to be able to deduce the appropriate application.

Data are still lacking for laboratory-based automated, high-throughput antigen tests. In this study, we evaluated the LIAISON SARS-CoV-2 antigen test (maximal throughput: 136 tests/h) for sensitivity and specificity. Besides that, the strength of this study is primarily (1) the size of the collective (1636 individuals), in which assay specificity was tested, (2) the comparison of the antigen test in the 196-individuals evaluation collective with two different PCR assays performed in two separate laboratories (currently, there is no internationally approved standardization for SARS-CoV-2 PCR testing), and (3) the fact that PCR and antigen testing were performed from the same sample, within 24 h of sample collection, and without prior freezing of the sample. Limitations of our study are: (1) Only the antigen-positive samples and 3 negative samples (samples with an AU/mL between 100 and 200 detected in the initial phase of the study) were checked by PCR in the 1636 individuals screening collective. (2) Patients with confirmed SARS-Cov-2 infection were sampled at any time during their hospitalization without direct correlation to symptom onset or knowledge of clinical courses.

LIAISON SARS-CoV-2 antigen test showed an overall diagnostic sensitivity of 40.2% and a specificity of close to 100% (Table 1), if the recommended cut-off of 200 AU/mL is applied. Exemplarily for the performance of RATs, the results of a recently published study by Osterman et al. [11] are compared with the LIAISON test. This study evaluated two rapid antigen tests for POCT use (SD Biosensor STANDARD™ F COVID-19 Ag FIA, and Roche Diagnostics SARS-CoV-2 Rapid Antigen Test) and is characterized by careful study design, the analysis of relatively large collectives (*n* = 360 and 386), and by an already completed peer-review process (most other evaluation studies are currently available as preprints). The authors found similar performance for both assays, which also matched the performance of many other RATs [6, 8, 11, 15], as well as our results for the LIAISON test. The overall clinical sensitivity was approx. 50.34% (Roche Diagnostics) and 45.41% (SD Biosensor), and the specificity approx. 97.67 and 97.78%, respectively. In the primary diagnosis of symptomatic and asymptomatic individuals, the diagnostic sensitivity was significantly higher (64.5% and 60.9%, respectively), because in these samples copy numbers (> 10^5^ copies/ml; Ct < 27) were significantly higher than that in the overall collective. Consistent with these observations, the WHO interim guidance from 11 September 2020 had already demanded a sensitivity of ≥ 80% and a specificity of ≥ 97% for RATs, but only for samples with a Ct < 25 (10^6^ copies/ml) in the RT-qPCR assay [9]. The LIAISON Test meets this requirement with a sensitivity of 91.2% and a specificity of 100% in samples with a Ct < 25. Consequently, compared with the RATs used in the Osterman study, the sensitivity of the LIAISON test is slightly lower at a cut-off of 200 AU/mL, while the specificity is rather higher. A more detailed analysis, however, reveals that this is exclusively caused by the choice of the cut-off: lowering the cut-off to 100 AU/mL results in a sensitivity of 49.7% (< 25 Ct 94.7%) and a specificity of 98.3%. In summary, we conclude that the quality of the high-throughput LIAISON test is the same as that of good RATs. However, the result of the LIASON test is available after 3 h at the earliest (sample transport, 2 h pre-incubation, 42 min assay run). Due to limited sensitivity, antigen tests provide a result that is reliable for only a short time (e.g. approx. 6 h). Therefore, prolonged transport and analysis time are relevant shortcomings of the LIAISON test compared to the RATs.

The observation of a patient with a negative LIAISON antigen result whose RT-qPCR result was highly positive the following day indicates what can also be expected from the sensitivity analysis: the occurrence of false-negative antigen test results in patients, who can be potentially highly infectious within a short period. False negative results are particularly relevant, when the aim, as in our case, is to screen patients without any symptoms of COVID-19 on hospital admission. Considering that patient collectives on hospital admission (including COVID-19 asymptomatic ones) usually show Ct values > 25 in SARS-CoV-19 RT-qPCR assays, even a limited number of patients with lower Cts, but rapid increase in viral load [16–18], are sufficient to infect staff and patients in a clinic. This is critical as the latter are predominantly elderly and patients with pre-existing conditions, who are at risk of severe COVID-19 disease. To roughly estimate how many false negatives are expected in our screening population, we calculated the false negatives within the 2 weeks of screening based on the following assumptions: (1) sensitivity of the LIAISON assay of 40.2–91.2%, specificity of 100% (negative predictive value of 99.9%), (2) 7-day incidence of 115.05–214.8 /100,000, and (3) average infectivity of patients of 7 days. This results in 1–2 false-negative patients, which corresponds well to the observed false-negative patient. However, assuming alternatively that 4 observed antigen-positive patients in the screening collective correspond to a sensitivity of 40.2% (Table 1) we would expect a much higher number (5) of false-negative results.

Considering these data, analysis time, and the recommendations of WHO and PEI, the LIAISON test is no reasonable alternative to PCR testing for asymptomatic patients on hospital admission (except for RT-qPCR being not available). The quality of the LIAISON test is the same as that of good RATs with the advantage of high throughput and the disadvantage of relatively long analysis time. The LIAISON test should therefore be used for the same applications as recommended for RATs [9, 19].

## Data Availability

The datasets generated and analyzed during the current study are available from the corresponding author on reasonable request.
